# Local Expression Dynamics of Various Adipokines during Induced Luteal Regression (Luteolysis) in the Bovine Corpus Luteum

**DOI:** 10.3390/ani13203221

**Published:** 2023-10-15

**Authors:** Granit Thaqi, Bajram Berisha, Michael W. Pfaffl

**Affiliations:** 1 Chair of Animal Physiology and Immunology, School of Life Sciences, Technical University of Munich, Weihenstephan, 85354 Munich, Germany; bajram.berisha@uni-pr.edu (B.B.); michael.pfaffl@tum.de (M.W.P.); 2 Department of Animal Biotechnology, Faculty of Agriculture and Veterinary, University of Prishtina, 10000 Prishtina, Kosovo

**Keywords:** corpus luteum, ovary function, induced luteolysis, adipokines, gene expression, cattle reproduction

## Abstract

**Simple Summary:**

Within the intricacies of the reproductive cycle, the regression of the corpus luteum (CL) plays a pivotal role. This temporary endocrine structure, formed post-ovulation, undergoes regression in the absence of pregnancy. The CL experiences rapid and profound changes in function and structure. This process, known as luteal regression or luteolysis, remains a cornerstone of the female reproductive system’s mechanics. Notably, within ovarian physiology, a domain of local regulation has been unveiled. Thus, our investigation set out to explore the enigmatic realm of locally produced adipokines and their receptor dynamics within the CL during experimentally induced luteolysis. Through meticulous examination at diverse stages of CL regression, we unveiled significant fluctuations in adipokine expression, unveiling their potential roles in orchestrating corpus luteum lysis or the resumption of cyclicity.

**Abstract:**

The study aimed to evaluate the mRNA expression levels of various local novel adipokines, including vaspin, adiponectin, visfatin, and resistin, along with their associated receptors, heat shock 70 protein 5, adiponectin receptor 1, and adiponectin receptor 2, in the corpus luteum (CL) during luteal regression, also known as luteolysis, in dairy cows. We selected Fleckvieh cows in the mid-luteal phase (days 8–12, control group) and administered cloprostenol (PGF analog) to experimentally induce luteolysis. We collected CL samples at different time points following PGF application: before treatment (days 8–12, control group) and at 0.5, 2, 4, 12, 24, 48, and 64 h post-treatment (n = 5) per group. The mRNA expression was measured via real-time reverse transcription polymerase chain reaction (RT-qPCR). Vaspin was characterized by high mRNA levels at the beginning of the regression stage, followed by a significant decrease 48 h and 64 h after PGF treatment. Adiponectin mRNA levels were elevated 48 h after PGF. Resistin showed upregulation 4 h post PGF application. In summary, the alterations observed in the adipokine family within experimentally induced regressing CL tissue potentially play an integral role in the local regulatory processes governing the sequence of events culminating in functional luteolysis and subsequent structural changes in the bovine ovary.

## 1. Introduction

Corpus luteum (CL) regression is a crucial process in the estrous cycle, where the CL, a temporary endocrine structure formed after ovulation, undergoes regression if pregnancy does not occur. Distinct from all other organs in the body, the ovaries undergo rapid and profound changes in function and structure [1,2]. These changes include cell proliferation, differentiation, and transformation, resulting in an ovulatory follicle and, hence, the CL’s formation, function, and regression in different species [3,4,5,6]. This series of events in ruminants is mainly controlled by gonadotropin surge and the dominance of estrogen produced by ovarian follicles and progesterone produced by the CL [7,8,9]. Nonetheless, cow fertility embodies a level of complexity that transcends the interplay of these widely recognized reproductive hormones. Instead, fertility is intricately connected with various additional components, including locally secreted mediators like growth factors, vasoactive peptides, cytokines, steroids, prostaglandins, and various other influential factors [10,11,12,13].

This luteal regression (luteolysis) is necessary for the estrous cycle to continue normally. The estrous cycle encompasses a sequence of reproductive occurrences that commence with estrus (heat) and end at the following estrus. Estrus is the period of sexual receptivity, commonly referred to as heat. CL regression is a tightly regulated process in the female reproductive system, primarily controlled by hormonal signals of progesterone (P4), luteinizing hormone (LH), prostaglandins (PG), gonadotropin-releasing hormone (GnRH), and follicle-stimulating hormone (FSH) [2,14,15,16]. These hormonal signals create an environment where the CL’s functional life span is limited if pregnancy does not occur. As these hormone levels decline, the CL undergoes structural and functional changes, ultimately leading to its regression [17,18,19]. The process involves cell death (apoptosis) and decreased P4 production, resulting in the decline in hormonal support for the uterus. It is important to note that while the overall process of CL regression is relatively consistent across species, there can be variations in the specific mechanisms and timings due to the unique reproductive physiology of each species. While the exact details can vary among species, a general overview of some critical molecular events that occur during CL regression includes hormonal regulation (P4 decline), prostaglandins (PGF2α), inflammatory mediators (cytokines, chemokines), apoptosis and cell death, angiogenesis, vascular changes, extracellular matrix remodeling, gene expression changes, and immune cell involvement [19,20,21,22,23].

Several cytokines, growth factors, and vasoactive peptides are involved in CL regression, and their roles can vary depending on the species, the stage of the estrous, and the specific cellular and molecular context [8,18,24,25,26,27]. Furthermore, a group of cytokines known as adipokines, mainly originating from adipose tissue, exerts influence over the function of various tissues through autocrine and paracrine mechanisms, while also playing a role in maintaining overall body homeostasis via an endocrine mode of action [28,29]. These adipokines encompass adiponectin, vaspin, visfatin, resistin, chemerin, leptin, and apelin, constituting a novel set of biochemical messengers [30,31]. Various other adipokines, such as resistin and visfatin, have been studied for their potential effects on reproduction and ovarian function. These adipokines can have pro-inflammatory effects and may influence factors like insulin resistance and inflammation, which could indirectly impact ovarian processes, including corpus luteum regression [32,33].

Over the past decade, a shred of growing evidence has illuminated the increasing role of adipose tissue in orchestrating energy homeostasis, metabolism, and reproduction and even modulating the inflammatory response [32,34]. The engagement of adipokines in the regression of CL remains a subject of continuous investigation, and our comprehension of their precise functions is still evolving. These adipokines are detectable in the later stages of CL development and serve a functional purpose in the ovarian physiology of mammals [30,35]. Furthermore, it has been established that the ovary is receptive to the effects of adipokines. Research has unveiled that these adipokines influence mechanisms linked to steroid synthesis and can modulate the production of crucial factors like P4 and PGs, which are pivotal regulators of CL longevity [36,37,38]. Consequently, adipokines have emerged as a class of factors that potentially significantly regulate CL function during luteal regression.

However, further research is needed to fully elucidate their functions and associations with other biochemical pathways, particularly in cattle. To identify more factors, our study aimed to investigate the ovarian expression levels and potential regulatory effects of adipokines at different time points in the CL during regression after experimentally inducing luteolysis. Specifically, we focused on the local adipokines mentioned above. Through our research, we hope to contribute to a better understanding of the role of adipokines in the CL regression stage, perhaps the most interesting and crucial stage for the reproductive performance of cattle.

## 2. Materials and Methods

### 2.1. Collection of Bovine Corpus Luteum during Induced Luteolysis

Forty multiparous Fleckvieh cows, housed at Veitshof, a research station of the TUM School of Life Sciences (Technical University of Munich, Freising, Germany), were implemented in the study. Within the mid-luteal phase, (on days 8–12), the control group, a subset of cycle-synchronized cows (n = 5 for each time point), received an intramuscular injection of 500 µg of cloprostenol, a PGF analog known as Estrumate (Intervet) for experimental luteolysis induction. Following this intervention, CL samples were collected through transvaginal ovariectomy. The animal trail was approved by the animal ethics committee located at the government of Upper Bavaria. Ovariectomies were conducted using the Chappat method (Chappat, 1993), also endorsed by Yart et al. (2012) [39,40]. Preceding the procedure, the cows received sedation through Rompun (10 mg, Bayer HealthCare) and Torbugesic (5 mg, Fort Dodge Animal Health). Post-cleansing, the dorsal vaginal wall and peritoneum were incised (approx. 3 cm) proximate to the cervix using “Krebs scissors”. Utilizing a “cow ovariectomy effeminator” (cow-specific ovariectomy device), ovarian pedicles were secured. Subsequently, ovaries were carefully extracted via the vaginal cavity. To mitigate the risk of rebleeding, the vascular pedicle of the ovary underwent compression in the effeminator for around 4 min. The vaginal incision was then sealed using 2–3 staples. A tampon soaked in penicillin was introduced into the vaginal canal to avert potential infections (this remained in place for 24 h). Cobactan (30 mL, Animal Health) was administered as a systemic antibiotic over 2 days, and the cows received Vetalgin (40 mL, Intervet) to preempt any postoperative discomfort. Hematocrit values were monitored within the subsequent 24 h post-operation to assess the risk of postoperative bleeding.

For CL sample collection, two distinct groups were established: control group samples (n = 5) were obtained between days 8 and 12 of the estrous cycle, and the experimental group samples were collected 0.5, 2, 4, 12, 24, 48, and 64 h after PGF application to obtain a comprehensive overview of potential cascade events. This sampling scheme is commonly employed in various experiments. Post-collection, the CL tissue samples were promptly frozen in liquid nitrogen and stored at −80 °C until RNA and protein extraction.

### 2.2. RNA Extraction and RT-qPCR

Dissected 50 mg CL tissues were subjected to total RNA extraction, employing a meticulous liquid–liquid extraction protocol. Specifically, the acid guanidinium thiocyanate–phenol–chloroform extraction method was utilized, using QIAzol^®^ Lysis Reagent (Qiagen, Hilden, Germany). The total RNA pellet was dissolved in 100 µL of nuclease-free water. To ensure the quality and quantity of the extracted RNA, absorbance readings were taken at 260 nm, and the 260/280 nm ratio was assessed using a NanoDrop^®^ ND-1000 Spectrophotometer (Thermo Fisher Scientific, Waltham, MA, USA). Notably, all samples exhibited exceptional RNA purity, with 260/280 ratios exceeding 2. Furthermore, the integrity of the RNA was rigorously evaluated through the utilization of an Agilent 2100 Bioanalyzer (Agilent Technologies, Santa Clara, CA, USA). This assessment generated a score termed the RNA Integrity Number (RIN), founded on the 28S to 18S rRNA ratio. This RIN score operates on a scale from 1 to 10, where a score of 1 signifies extensive RNA degradation, while a score of 10 reflects an impeccable RNA extract. The depicted samples consistently exhibited a RIN surpassing 5 throughout our experimental proceedings. Primer pairs and hydrolysis oligonucleotide probes were designed for three housekeeping genes (*ubiquitin A52*, *ubiquitin C*, and *cyclophilin A*) and target genes (*vaspin*, *heat shock 70 kDa protein 5-HSPA5*, *adiponectin*, *adipoR1*, *adipoR2*, *visfatin*, and *resistin*). This design process was executed using the Primer3 program (version 4.1.0) in conjunction with databases sourced from Ensembl (EMBL-EBI, Cambridge, UK). Additionally, NetPrimer (Premier Biosoft, San Francisco, CA, USA) was employed to ensure primer integrity for a comprehensive analysis aimed at averting any homologies and predicting potential template secondary structures. The sequence similarities of both primers and probes were further scrutinized using the Standard Nucleotide BLAST program (NCBI), ensuring that all primers exhibited specificity to the target sequences (Table 1). The primers were of HPLC grade and ordered from Biomers.net (accessed on 2 February 2022) GmbH (Ulm, Germany).

The isolated RNA underwent a precise dilution process to attain the desired 10 ng/µL concentration, tailored explicitly for quantifying mRNA transcripts using RT-qPCR. For this purpose, we utilized the Luna^®^ Universal Probe One-PCR system, provided by New England BioLabs Inc. (Ipswich, MA, USA), to conduct one-step real-time quantification in a 10 µL reaction volume. This reaction mixture consisted of 5 µL of Luna Universal Probe One-Step Reaction Mix (New England BioLabs Inc., Ipswich, MA, USA), 0.5 µL of Luna WarmStart RT Enzyme Mix, 0.4 µL each of forward and reverse primers designed for the targeted sequence (each at a concentration of 10 µM), 0.2 µL of the corresponding hydrolysis probe, 3.0 µL of the RNA sample, and 0.5 µL of nuclease-free water. The following thermal cycling protocol was applied using a Rotor-Gene Q thermal cycler (model 5-Plex HRM, Qiagen). Initially, a reverse transcription (RT) step was performed at 55 °C for 10 min, followed by initial denaturation at 95 °C. Subsequently, 45 cycles of denaturation at 95 °C for 10 s and extension at 60 °C for 30 s were carried out. To verify the quality and ensure the expected length of the RT-qPCR products aligned with the target sequence, electrophoresis was conducted using 2% agarose gel (VWR International, Darmstadt, Germany). It is worth noting that the entire RT-qPCR workflow adhered meticulously to the MIQE (Minimum Information for Publication of Quantitative Real-Time PCR Experiments) guidelines [35].

### 2.3. Determination of Relative mRNA Expression

The analysis process was initiated by extracting the Cq values, representing the threshold data, by employing the Rotor-Gene Rotor Q Software 2.3.1 (Qiagen, Hilden, Germany) and utilizing the comparative quantitation method. Levels of mRNA expression were normalized against the geometric mean derived from three consistently expressed reference genes: *cyclophilin A* (*PPIA*), *ubiquitin A52*, and *ubiquitin C*. In-depth data analysis, utilizing the geNorm and NormFinder algorithms within the GenEX software (version 7, MultiD; Sweden), revealed the geometric mean as the most robust and reliable normalizer. The geNorm algorithm assessed the stability of prospective reference genes by comparing their average pairwise variation with other reference genes. Those with the least pairwise variation were identified as the most stable, making them the preferred choice for normalizing gene expression data. Conversely, the NormFinder algorithm evaluated the stability of each potential reference gene, considering both intragroup and intergroup variations. Genes with lower stability values were considered more dependable and were recommended for normalizing gene expression data [41,42].

Subsequently, after the normalization process utilizing the geometric mean of the housekeeping genes, the next step involved comparing the normalized expression differences using the ∆∆Cq method between two distinct sample groups: the “treatment group” and the non-treated “control group” [43]. Relative gene expression was calculated by comparing the treatment group to the control group to determine the variation in mRNA expression, presented as fold change regulation. The set of cycling cows in the mid-luteal stage (on days 8–12) was deemed the control group and was assigned a value of 1.0. All relative mRNA expression changes are presented as x-fold regulation, accompanied by ∆Cq SEM per group. The relative gene expression was calculated by juxtaposing the treatment group with the control group to ascertain the variance in mRNA expression, depicted as fold change regulation. All relative mRNA expression change outcomes are presented as x-fold regulation, accompanied by ∆Cq SEM per group.

### 2.4. Statistical Analysis

In this research, we retrieved CLs from cycling dairy cows during two distinct phases: days 8–12 of the estrous cycle, constituting the control group, and at various time intervals after the application of PGF—namely, 0.5, 2, 4, 12, 24, 48, and 64 h post-treatment (with n = 5 samples per group). The data are presented as ΔCq means, meticulously accompanied by the standard error of the mean (SEM). A comprehensive analysis was undertaken to evaluate the statistical significance of observed alterations. It employed a one-way ANOVA, followed by Tukey’s HSD and LSD for multifaceted comparison assessments, through IBM SPSS (New York, NY, USA). Notably, changes were deemed statistically significant if they demonstrated a *p*-value of less than 0.05.

## 3. Results

**Figure 1:**
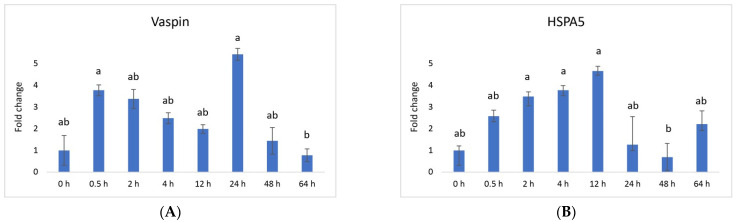
Relative mRNA expression of (**A**) vaspin and its receptor (**B**) HSPA5 (heat shock protein 5) and in the CL after experimentally induced luteolysis (n = 5). Threshold data were normalized to the geometric mean of the three reference genes (*cyclophilin A*, *ubiquitin A52*, and *ubiquitin C*). The data are plotted as fold change ± SEM of group ∆Cq. The non-treated group of cows on days 8–12 of the cycle was used as the control group (0 h). The different significance levels are indicated by different superscripts (*p* < 0.05).

**Figure 2:**
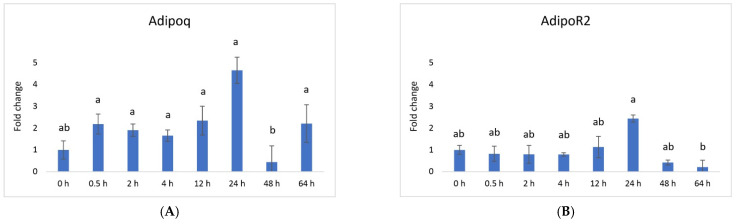
Relative mRNA expression of (**A**) adiponectin and its receptor (**B**) adiponectin receptor 2 in the CL after experimentally induced luteolysis (n = 5). Threshold data were normalized to the geometric mean of the three reference genes (*cyclophilin A*, *ubiquitin A52*, and *ubiquitin C*). The data are plotted as fold change ± SEM of group ∆Cq. The non-treated group of cows on days 8–12 of the cycle was used as the control group (0 h). The different significance levels are indicated by different superscripts (*p* < 0.05).

**Figure 3:**
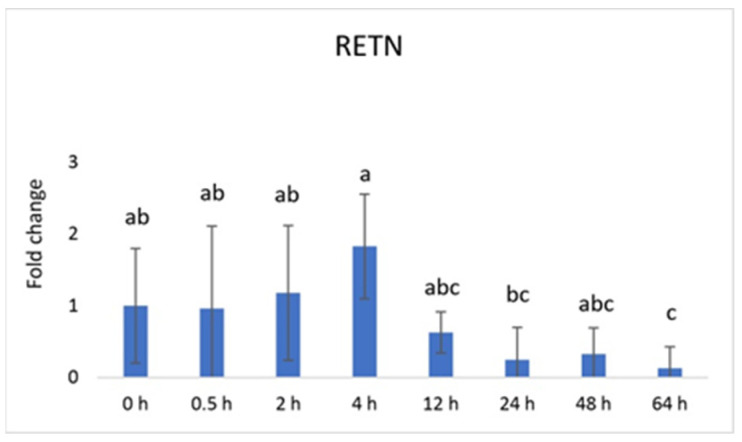
Relative mRNA expression of resistin (RETN) in the CL after experimentally induced luteolysis (n = 5). Threshold data were normalized to the geometric mean of the three reference genes (*cyclophilin A*, *ubiquitin A52*, and *ubiquitin C*). The data are plotted as fold change ± SEM of group ∆Cq. The non-treated group of cows on days 8–12 of the cycle was used as the control group (0 h). The different significance levels are indicated by different superscripts (*p* < 0.05).

Vaspin exhibited a distinctive mRNA expression pattern, with high levels observed at 0.5 h and 24 h after PGF application (Figure 1A). However, there was a significant decrease at 64 h. The *HSPA5* gene, responsible for producing the vaspin receptor, displayed abundance at 2, 4, and 12 h, gradually declining after that, reaching its lowest level at 48 h (Figure 1B). Adiponectin’s mRNA levels remained consistently expressed across all time points, with a downregulation noted at 48 h (Figure 2A). In contrast, the expression of adiponectin receptor 1 remained at low levels without significant changes. Meanwhile, after PGF application, adiponectin receptor 2 (AdipoR2) exhibited an upregulation specifically at the 24 h time point (Figure 2B). Visfatin showed no significant expression changes. Notably, resistin (RETN) demonstrated elevated levels at 0.5 h, 2 h, 4 h, and 12 h, followed by a substantial decrease at 64 h (Figure 3). Generally, we observed significant changes between treatment groups, while no significant changes were found between the control group and the treated groups.

## 4. Discussion

CL regression is a natural process in the female reproductive system, primarily in response to the absence of pregnancy. As mentioned earlier, the decrease in circulating P4 and other hormones like LH is a central event in initiating CL regression [44]. Prostaglandin F2α (PGF2α) is critical in luteolysis, triggering vasoconstriction and reduced blood flow to the CL. PGF2α is released by the uterus and locally acts on receptors in the CL [45]. In response to PGF2α and other factors, inflammatory mediators like cytokines and chemokines are produced [24]. These molecules contribute to the recruitment of immune cells, such as macrophages, which play a role in clearing cellular debris during regression [46,47]. 

Moreover, CL regression also includes the activation of various apoptotic pathways within the CL and a decrease in blood supply to the CL as a result of both vasoconstriction due to PGF2α and changes in the expression of local growth factors like angiogenic, luteolytic, and extracellular matrix remodeling factors [21,48,49,50]. These gene expression changes are responsible for reducing P4 levels and increasing pro-apoptotic gene activity. Additionally, immune cells, particularly macrophages, enter the regressing CL and help clear away apoptotic cells and tissue debris [46,47,49,51,52,53,54]. 

Exploring novel cytokine families like adipokines and their role in this process holds significant promise. Adipokines exhibit dual characteristics—(1) pro-inflammatory (resistin, visfatin) and (2) anti-inflammatory (vaspin, adiponectin)—making them potential players in inflammation within the CL. Regrettably, the existing data on the impact of adipokines on CL regression remain scarce in most species, constraining the extent to which their role can be understood. Our research is the first effort to demonstrate the expression of adipokines during experimentally induced CL regression. While studies demonstrate the indirect influence of adipokines on local ovarian function, their direct involvement in CL regression remains a topic requiring further substantiation [23,55,56].

Adipokines comprise a collection of bioactive peptides and proteins released from adipose tissue that promote molecule signaling communication through myriad pathways within the body. Notably, specific adipokines such as tumor necrosis factor-alpha (TNF-α) and interleukin-6 (IL-6) are known to instigate the nuclear factor kappa-light-chain-enhancer of activated B cells (NF-κB) pathway, integral to inflammation and immune activity. Leptin and resistin, among other adipokines, activate the JAK/STAT pathway linked to inflammation and immunity. Adiponectin, another adipokine, spurs AMP-activated protein kinase (AMPK) activity, which is pivotal in governing cellular metabolism and energy equilibrium. Additionally, adiponectin, leptin, and resistin influence insulin signaling, which is crucial in glucose and lipid metabolism. Moreover, adiponectin and leptin can set mitogen-activated protein kinases (MAPKs) in motion, which are pivotal for cell proliferation, differentiation, and apoptosis. Essentially, adipokines’ signaling via these routes intricately manages glucose and lipid metabolism, inflammation, immune response, and other vital physiological processes within the body [55,57]. 

Vaspin is an adipokine family member that was first detected in white adipose tissue in 2005. It has been strongly linked with glucose metabolism, insulin resistance, obesity, and inflammation [58]. Vaspin can bind to the cell surface of protein GRP78, also known as heat shock protein family member 5 (HSPA5) or binding immunoglobulin protein. Both vaspin and HSPA5 expressions are found in different ovarian structures. These structures include ovarian follicles, oocytes, and the CL. Research has suggested that their presence in the porcine ovary may play a role in processes such as angiogenesis, proliferation, and luteal cell apoptosis. [14,30]. In our study, vaspin demonstrated high mRNA levels at 0.5 h and 24 h after prostaglandin F (PGF) application, indicating potential involvement in the luteolysis process (Figure 1A). Meanwhile, HSPA5 displayed peak abundance at 2, 4, and 12 h, gradually declining and reaching its lowest point at 48 h (Figure 1B). Adiponectin, a cytokine secreted by the adipose tissue, plays a pivotal role in regulating various metabolic processes, such as glucose levels and lipid metabolism. These metabolic processes are regulated through the action of two receptors, adiponectin receptor 1 (AdipoR1) and adiponectin receptor 2 (AdipoR2). Adiponectin’s mRNA levels remained consistently expressed across all time points, with a downregulation at 48 h (Figure 2A). Additionally, adiponectin has been suggested to influence angiogenic factors in developing porcine CL [59]. The expression of adiponectin receptor 1 remained at low levels without significant changes. In contrast, after PGF application, adiponectin receptor 2 exhibited upregulation at the 24 h time point (Figure 2A). Resistin is a peptide hormone rich in cysteine derived from adipose tissue. It was initially identified in mice as a protein that appeared to induce insulin resistance, hence its name, “resistin”. Interestingly, resistin displayed elevated levels at 0.5 h, 2 h, 4 h, and 12 h, followed by a decrease at the 64 h mark (Figure 3). Studies have reported that resistin can elevate estradiol (E2) levels, reduce P4 levels, and regulate several pathways, including those associated with steroidogenic acute regulatory protein (STAR), cholesterol side-chain cleavage enzyme, 3β-hydroxysteroid dehydrogenase, and estrogen synthetase. These effects are brought about through the activation of protein kinase A and mitogen-activated protein kinase 1 [37,60]. Visfatin is another adipokine that plays a role in various physiological processes, including metabolism and inflammation. It was initially identified as a protein secreted by adipose tissue but is also produced by other cells, including immune cells. Interestingly, in our study, visfatin did not exhibit significant expression levels. It has also been implicated in the regulation of immune responses and inflammation, serving as a pro-inflammatory adipokine by augmenting the production of inflammatory cytokines by monocytes and leukocytes, in addition to activating nuclear factor kappa B (NFKB) signaling [61,62].

It is worth noting that the detection and histological localization of the mature proteins and the receptors of the studied adipokine family within bovine species poses a challenge due to the absence of commercially available antibodies. Developing precise and dependable antibodies is essential for enhancing our comprehension of adipokines across various molecular levels. This, in turn, will aid in assessing their expression potency and precise tissue localization, enabling us to speculate about their potential role in reproductive function and shed light on their underlying mechanisms. 

Our research provides data that push our comprehension and identify the intricate mechanisms and signaling involved in dairy cattle reproduction. Our discoveries provide valuable perspectives into how adipokines are locally regulated within the bovine CL, enriching our insight into the complex interaction between adipokines and reproductive physiology. Furthermore, we underline the necessity of investigating additional factors that might impact the interplay of reproductive mechanisms in dairy cattle.

## 5. Conclusions

Our findings highlight distinct mRNA expression patterns of adipokines within the CL during its regression stage, underscoring their potential roles in regulating CL functionality. Furthermore, the varying up- or downregulation of adipokine mRNA levels across different stages underscores their intricate regulatory impacts. The time-specific expression of adipokines and their corresponding receptors within the CL strongly implies their active involvement in local mechanisms governing CL activity. In conclusion, our current investigation firmly establishes the expression of all studied adipokines within bovine CL mRNA during the experimental induced regression stages. However, in-depth inquiries are necessary to fully elucidate the precise regulations and underlying mechanisms driving the diverse local effects of these adipokines within the CL. Comprehending the precise roles that adipokines play in CL function can pave the way for targeted therapies to enhance fertility and reproductive capabilities in dairy cows.

## Figures and Tables

**Table 1:** Primer sequence, probe sequences, RT-qPCR product length, and Ensembl transcript ID of amplified target genes: *vaspin*, *heat shock protein 5* (*HSPA5*), *adiponectin*, *adiponectin receptor 1* (*AdipoR1*), *adiponectin receptor 2* (*AdipoR2*), and *resistin*; and reference genes: *ubiquitin A52* (*UBA52*), *ubiquitin C* (*UBC*), and *cyclophilin A* (*PPIA*).

Genes	Sequence of Nucleotides *	Amplicon Size [bp]	Ensembl Transcript ID
*PPIA*	For-5′-CTGAGCACTGGAGAGAAAGGA-3′	116	ENSBTAT00000015924.5
	Rev-5′-GACTTGCCACCAGTACCATT-3′		
	Hyb-5′-TCCGGGATTTATGTGCCAGGGTGGT-3′		
*UBA52*	For-5′-GGCTGATCTTCGCTGGCA-3′	124	ENSBTAT00000010176.3
	Rev-5′-CGGAGGGAAGGCTCGATG-3′		
	Hyb-5′-TGGAGGATGGCCGCACTCTGTCAGA-3′		
*UBC*	For-5′-GACCGGGAGTTCAGTCTTCG-3′	133	ENSBTAT00000046011.4
	Rev-5′-TTCTCGATGGTGTCACTGGG-3′		
	Hyb-5′-TGTGTTCGCTGCTGACACCACCACT-3′		
*Vaspin*	For- 5′-TACCAGAGCAACTTCACGGC-3′	146	ENSBTAT00000045204.4
	Rev-5′-GTCAACCTGGGCACAAACAC-3′		
	Hyb-5′-TGAAGCAAGTGGAGCAAGCCCTGGG-3′		
*HSPA5*	For-5′-TTTCTGCCATGGTTCTCACT-3′	125	ENSBTAT00000057533.3
	Rev-5′-ATCTTTGGTTGCCTGGCGTT-3′		
	Hyb-5′-AGGAAACTGCTGAGGCTTATTTGGGA-3′		
*Adiponectin*	For-5′-GTGAGAAGGGTGAGAAAGGAGA-3′	136	ENSBTAT00000077795.1
	Rev-5′-GCACTTTCTCCAGGTTCTCCC-3′		
	Hyb-5′-GAGGCTTTCCAGGAACCCCAGGCAG-3′		
*AdipoR1*	For-5′-CCACACTGTCTACTGTCATTCA-3′	150	ENSBTAT00000040283.5
	Rev-5′-GAGAGGTAGATGAGCCGAGG-3′		
	Hyb-5′-TGCTGATCATGGGGAGCTTCGTGCC-3′		
*AdipoR2*	For-5′-GGCGTCTGTCCTTTCTTCCT-3′	110	ENSBTAT00000011923.6
	Rev-5′-CTTGCAGGAGAGGGGACATG-3′		
	Hyb-5′-GGAGCGTGAGTGCGATGATGGAGCA-3′		
*Resistin*	For-5′-CAGTCGCTGTGCCCCATAG-3′	91	ENSBTAT00000006189.3
	Rev-5′-GGCCAATGATCCTTACTGCC-3′		
	Hyb-5′-TGAGAAGATCCAGGAGGTCACCACC-3′		
*Visfatin*	For-5′-TCGAAGGGCTACAAGTTGCT-3′	143	ENSBTAT00000020608.4
	Rev-5′-GCTCCACCAGAACCAAAGGA-3′		
	Hyb-5′-ACAAGAGATTGTGGAAGGCATGAAGCA-3′		

* For, forward primer; Rev, reverse primer; Hyb, hybridization probe.

## Data Availability

Further data supporting this study’s findings are available from the corresponding author upon reasonable request.
